# Protective effect of carnosic acid on bisphenol A‐induced neuroinflammation and oxidative stress in HMC3 microglia cells

**DOI:** 10.1002/alz70855_098116

**Published:** 2025-12-23

**Authors:** Chia‐Wen Tsai, Han‐Ting Wu, Chun‐Huei Liao, His‐Yun Huang, Ya‐Chen Shih

**Affiliations:** ^1^ China Medical University, Taichung, Taichung, Taiwan

## Abstract

**Background:**

Bisphenol A (BPA) is an endocrine‐disrupting chemical commonly used in polycarbonate plastic production (Mhaouty‐Kodja et al., 2024). Studies indicate that BPA activates microglial cells, leading to potential neuroinflammation (Ouyang et al., 2024). Carnosic acid (CA), a phenolic diterpene from rosemary (*Rosmarinus officinalis* L.), has potent antioxidant and neuroprotective properties (Hsu et al., 2024). This study investigated the protective effects of carnosic acid (CA) against BPA‐induced neuroinflammation and oxidative stress in human HMC3 microglia cellsand C57BL/6J male mice.

**Method:**

*In vitro* experiments, cells were treated with 20 nM BPA and 1 μM CA for 3 h to investigate the effect of neuroinflammation and oxidative stress. Protein levels of inflammasome components, antioxidant enzymes, and transcription factors were assessed. We further used *in vivo* model to confirm the antioxidant capacity of CA. Mice were divided into four groups: control (olive oil 0.01 mL /g weight), BPA (50 μg/kg BPA), BLCA (50 μg/kg BPA + 5 mg/kg CA), BHCA (50 μg/kg BPA + 20 mg/kg CA). The antioxidant capacity of CA was assessed.

**Result:**

CA treatment improved the BPA‐induced inflammasome proteins, including NLRP3, cleaved caspase‐1, GSDMD‐N, and IL‐1β. Moreover, CA improved the BPA‐induced fluorescence expression of reactive oxygen species (ROS). CA also reversed the antioxidant enzymes, transcription factor Nrf2, and UDP glucuronosyltransferase (UGT) 1A1 protein reduced by BPA. *In vivo* experiments, CA treatment restored BPA‐induced the reduction of proteins in UGT1A1 and antioxidant enzymes in the striatum. The antioxidant capacity of CA was reduced in cells pretreated with *L*‐buthionine‐sulfoximine (BSO), a GSH synthesis inhibitor.

**Conclusion:**

These findings indicated that the neuroprotection of CA could reverse BPA‐induced oxidative stress and neuroinflammation by regulating inflammasome proteins and antioxidant enzymes in HMC3 microglial cells.

